# Clinical and virological course of patients with coronavirus disease 2019 in Jiangsu province, China: a retrospective, multi-center cohort study

**DOI:** 10.1186/s12985-021-01615-y

**Published:** 2021-07-14

**Authors:** Rui Huang, Li Zhu, Leyang Xue, Xuebing Yan, Jian Wang, Songping Huang, Biao Zhang, Tianmin Xu, Fang Ji, Chunyang Li, Fang Ming, Yun Zhao, Yang Li, Juan Cheng, Yinling Wang, Huaping Shao, Shuqin Hong, Kang Chen, Xiang-an Zhao, Dawen Sang, Lei Zou, Haiyan Zhao, Xinying Guan, Xiaobing Chen, Biyun Xu, Juan Xia, Yuxin Chen, Xiaomin Yan, Jie Wei, Jiacheng Liu, Longgen Liu, Chuanwu Zhu, Chao Wu

**Affiliations:** 1 Department of Infectious Diseases, Nanjing Drum Tower Hospital, The Affiliated Hospital of Nanjing University Medical School, Nanjing, China; 2 Department of Infectious Diseases, The Affiliated Infectious Diseases Hospital of Soochow University, Suzhou, China; 3 Department of Critical Medicine, Huai’an No. 4 People’s Hospital, Huai’an, China; 4 Department of Infectious Diseases, Affiliated Hospital of Xuzhou Medical University, Xuzhou, China; 5 Department of Infectious Diseases, Nantong Third People’s Hospital, Nantong University, Nantong, China; 6 Department of Quality Control Office, Huai’an No. 4 People’s Hospital, Huai’an, China; 7 Department of Infectious Diseases, The Third People’s Hospital of Changzhou, Changzhou, China; 8 Department of Infectious Diseases, The Third People’s Hospital of Yangzhou, Yangzhou, China; 9 Department of Infectious Diseases, Taizhou People’s Hospital, Taizhou, China; 10 Department of Infectious Diseases, Yancheng Second People’s Hospital, Yancheng, China; 11 Department of Infectious Diseases, The People’s Hospital of Suqian, Suqian, China; 12 Nursing Department, Huai’an No. 4 People’s Hospital, Huai’an, China; 13 Department of Tuberculosis, The Third People’s Hospital of Changzhou, Changzhou, China; 14 Department of Gastroenterology, Northern Jiangsu People’s Hospital, Clinical Medical College of Yangzhou University, Yangzhou, China; 15 Department of Neurology, The Affiliated Hospital of Kangda College of Nanjing Medical University, The First People’s Hospital of Lianyungang, Lianyungang, China; 16 Department of Emergency, The Affiliated Hospital of Kangda College of Nanjing Medical University, The First People’s Hospital of Lianyungang, Lianyungang, China; 17 Department of Biostatistics, Nanjing Drum Tower Hospital, The Affiliated Hospital of Nanjing University Medical School, Nanjing, China; 18 Department of Laboratory Medicine, Nanjing Drum Tower Hospital, The Affiliated Hospital of Nanjing University Medical School, Nanjing, China; 19 Department of Infectious Diseases, Nanjing Drum Tower Hospital Clinical College of Nanjing Medical University, Nanjing, China; 20 Department of Infectious Diseases, Nanjing Drum Tower Hospital Clinical College of Traditional Chinese and Western Medicine, Nanjing University of Chinese Medicine, Nanjing, China

**Keywords:** Coronavirus disease 2019, SARS-CoV-2, Viral, Clearance

## Abstract

**Background:**

The clinical and virological course of patients with coronavirus disease 2019 (COVID-19) are lacking. We aimed to describe the clinical and virological characteristics of COVID-19 patients from 10 designated hospitals in 10 cities of Jiangsu province, China. The factors associated with the clearance of SARS-CoV-2 were investigated.

**Methods:**

A total of 328 hospitalized patients with COVID-19 were retrospectively recruited. The epidemiological, clinical, laboratory, radiology and treatment data were collected. The associated factors of SARS-CoV-2 clearance were analyzed.

**Results:**

The median duration of hospitalization was 16.0 days (interquartile range [IQR] 13.0–21.0 days). On multivariate Cox regression analysis, age > 60 years (hazard ratio [HR] 0.643, 95% confidence interval [CI] 0.454–0.911, *P* = 0.013) was associated with the delayed SARS-CoV-2 clearance, while the atomized inhalation of interferon α-2b could improve the clearance of SARS-CoV-2 (HR, 1.357, 95% CI 1.050–1.755, *P* = 0.020). Twenty-six (7.9%) patients developed respiratory failure and 4 (1.2%) patients developed ARDS. Twenty (6.1%) patients were admitted to the ICU, while no patient was deceased.

**Conclusions:**

Our study found that age > 60 years was associated with the delayed SARS-CoV-2 clearance, while treated with atomized inhalation of interferon α-2b could promote the clearance of SARS-CoV-2.

**Supplementary Information:**

The online version contains supplementary material available at 10.1186/s12985-021-01615-y.

## Background

The outbreak of coronavirus disease 2019 (COVID-19) in Wuhan, China, caused by the severe acute respiratory syndrome coronavirus 2 (SARS-CoV-2) [1], continues to spread among humans. As of May 14, 2021, 160,686,749 confirmed cases and 3,335,948 deaths have been reported [2]. The clinical spectrum of SARS-CoV-2 infection presents from asymptomatic infection to severe pneumonia with fatal outcomes [3]. Although the epidemiological, radiologic and clinical characteristics of COVID-19 patients have been reported in several studies [4–6], the details of clinical and virological course of patients with COVID-19 are lacking. Zhou et al. described the clinical course and clearance of SARS-CoV-2 during hospitalization of COVID-19 patients with a definite clinical outcome in Wuhan [7]. However, several studies have demonstrated that the characteristics of patients outside of Wuhan were different from those initially reported patients in Wuhan [8–10]. Furthermore, the factors which affect the clearance of SARS-CoV-2 have not been identified.

In this multi-center study, we described the clinical and virological characteristics of patients with COVID-19 and explored the factors associated with the clearance of SARS-CoV-2 in Jiangsu province, China.

## Methods

### Patients

Between January 18, 2020 and February 29, 2020, patients confirmed with COVID-19 were retrospectively recruited from ten designated hospitals in 10 cities of Jiangsu province, China. The last follow-up date was March 10, 2020.

COVID-19 was confirmed by quantitative real-time reverse transcription polymerase chain reaction (RT-PCR) test using the same protocol described previously [11]. Throat swab specimens of all confirmed patients was collected on admission and during hospitalization. Laboratory tests were carried out in these designated hospitals and local Center for Disease Control and Prevention.

### Procedures

We collected and reviewed all the medical records of confirmed COVID-19 patients in each medical center. Data of patients regarding the epidemiological, clinical, laboratory, radiology and treatment were collected. The criteria of acute respiratory distress syndrome (ARDS) was based on the corresponding guidelines [12]. The management strategies and discharge criteria of COVID-19 in different hospitals were in accordance with the guidelines by the Chinese National Health Commission (Trial Version 5) [13]. The frequency of RT-PCR testing was determined by specific clinicians who were responsible for the management of patients according to the disease progression. The time of SARS-CoV-2 RNA negative was defined as two consecutively negative SARS-CoV-2 nucleic acid by RT-PCR test separated by at least 1 day. The days of viral clearance were calculated by the time of SARS-CoV-2 RNA negative minus the time of initial symptoms.

All patients were confirmed by throat swab samples tested by a RT-PCR in accordance with the protocol by the World Health Organization [14].

### Statistical analysis

The median and interquartile range (IQR) were used to present the continuous variables. Categorical variables were described as the counts and percentages. The independent group t tests (normal distribution) and Mann–Whitney *U* (non-normal distribution) were used to compare continuous variables between groups. Chi-square or Fisher’s exact test was used to compare the categorical variables. Cox regression was used to analyze the associated factors of SARS-CoV-2 clearance. Variables having *P* values < 0.1 in the univariate analysis were further used for a multivariate Cox regression analysis. Accumulative clearance of SARS-CoV-2 were generated using Kaplan–Meier method. Log-rank test was used to test the equivalences of the survival curves. *P* values for multiple comparisons was not corrected in our study. *P* < 0.05 was considered to be statistically significant. SPSS version 22.0 software (SPSS Inc., Chicago, IL, United States) was used for the data analysis.

## Results

### Demographic and epidemiologic characteristics

**Fig. 1:**
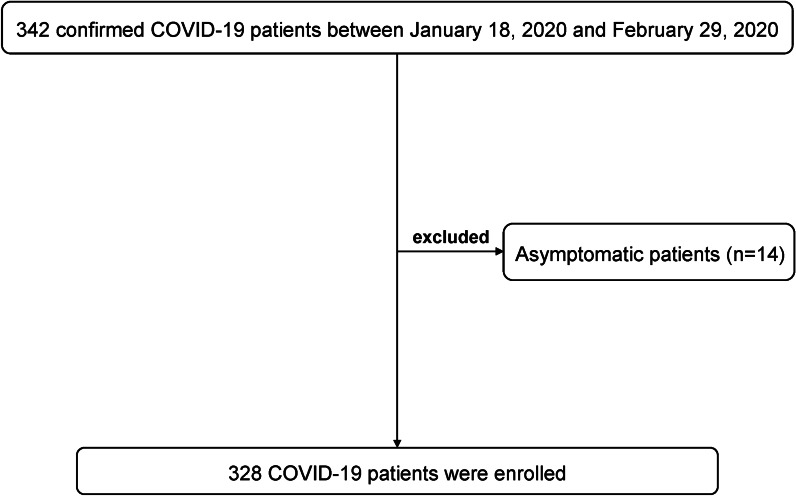
Flow diagram describing the selection of study population

**Fig. 2:**
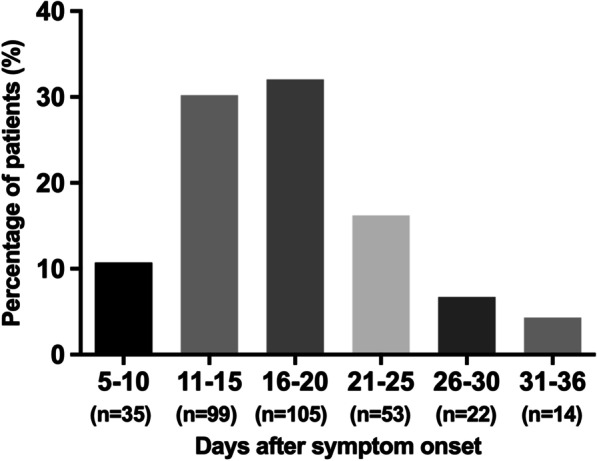
The distribution of viral clearance days of COVID-19 patients

A total of 342 hospitalized patients during January 18, 2020 to February 29, 2020 who were confirmed as SARS-CoV-2 infection were identified. Fourteen asymptomatic patients were also excluded. Finally, 328 COVID-19 patients were included for the analysis (Fig. 1). All patients were discharged before March 10, 2020. The median age of the patients was 45.0 (IQR 33.0–55.0) years (Table 1). Most (84.8%) of the patients were aged < 60 years. 176 (53.7%) patients were male. The median body mass index (BMI) was 24.2 (IQR 22.2–26.2) kg/m^2^. 88 (26.8%) patients had at least one underlying comorbidity, including hypertension (50 [15.2%]), type 2 diabetes (27 [8.2%]), chronic lung diseases (12 [3.7%]), chronic liver diseases (9 [2.7%]), cardiovascular diseases (6 [1.8%]), and malignant tumors (5 [1.5%]). The median time from symptom onset to admission was 5.0 (IQR 2.0–8.0 days) days. The median duration of hospitalization was 16.0 (IQR 13.0–21.0) days. All patients cleared the SARS-CoV-2 during the study period and the median duration of viral clearance after COVID-19 onset was 17.0 (IQR 13.0–21.0) days. The distributions of viral clearance days among these patients were presented in Fig. 2.

**Table 1:** Demographic and epidemiologic characteristics of patients with coronavirus disease 2019

Variables (n [%] or median [IQR])	All patients (n = 328)
Age (year)	45.0 (33.0, 55.0)
Age range	
< 60	278 (84.8)
≥ 60	50 (15.2)
Gender	
Male	176 (53.7)
Female	152 (46.3)
BMI (kg/m^2^)*	24.2 (22.2, 26.2)
Comorbidities	
Any comorbidity	88 (26.8)
Hypertension	50 (15.2)
Type 2 diabetes	27 (8.2)
Chronic lung diseases	12 (3.7)
Chronic liver diseases	9 (2.7)
Cardiovascular diseases	6 (1.8)
Malignant tumors	5 (1.5)
Smoking history	23 (7.0)

*PCR* polymerase chain reaction, *IQR* interquartile range, *BMI* body mass index

*Available for 292 patients

### Clinical features and laboratory abnormalities

Fever (237 [72.3%]) and cough (200 [61.0%]) were the most common symptoms (Table 2). 85 (25.9%) patients showed leukopenia and 99 (30.2%) patients had lymphopenia on admission. Chest CT scans were performed at the time of admission, 93.3% of the patients had abnormal results. The most common patterns on chest CT images were ground-glass opacity (73.2%). No CT abnormality was found in 22 (6.7%) patients.

**Table 2:** Clinical Characteristics and laboratory findings of patients with coronavirus disease 2019

Variables (n [%] or median [IQR])	All patients (n = 328)
*Onset signs and symptoms*	
Fever	237 (72.3)
Cough	200 (61.0)
Fatigue	69 (21.0)
Sore throat	34 (10.4)
Muscle ache	35 (10.7)
Shortness of breath	27 (8.2)
Headache	20 (6.1)
*Laboratory findings*	
WBC (× 10^9^/L)*	4.8 (3.8, 6.1)
Decreased	85 (25.9)
Lymphocyte (× 10^9^/L)^#^	1.2 (0.9, 1.6)
Decreased	99 (30.2)
Hb (g/L)	138.0 (128.0, 151.0)
PLT (× 10^9^/L)	174.5 (140.0, 219.3)
ALT (U/L)	25.0 (19.0, 35.3)
LDH (U/L)	243.0 (179.3, 390.3)
ALB (g/L)	40.1 (37.1, 43.3)
Cr (μmol/L)	64.0 (52.0, 78.0)
PT (s)	12.8 (12.0, 13.4)
D-Dimer (mg/L)	0.2 (0.1, 0.4)
*Chest CT findings*	
No pneumonia	22 (6.7)
Unilateral pneumonia	49 (14.9)
Bilateral pneumonia	257 (78.4)
Ground glass opacity	240 (73.2)
Time from symptom onset to admission (days)	5.0 (2.0, 8.0)

*PCR* polymerase chain reaction, *IQR* interquartile range, *WBC* white blood cells, *Hb* hemoglobin, *PLT* platelet, *TB* total bilirubin, *ALT* alanine transaminase, *LDH* lactate dehydrogenase, *ALB* albumin, *Cr* creatinine, *PT* prothrombin time

*The normal ranges of WBC were 4–10 × 10^9^/L or 3.5–9.5 × 10^9^/L in different hospitals and decreased means below the lower limit of the normal range

^#^The normal ranges of lymphocyte were 0.8–4 × 10^9^/L or 1.1–3.2 × 10^9^/L in different hospitals and decreased means below the lower limit of the normal range

### Complications, treatment and outcomes

Oxygen therapy was administered in 203 (61.9%) of patients and mechanical ventilation in 15 (4.6%) of patients. Majority of the patients (78.0%) received empirical antibiotic therapy. Glucocorticoids were given to 84 (25.6%) patients. Most of the patients received at least one antiviral drug (atomized inhalation of interferon α-2b, 55.2%; lopinavir/ritonavir, 74.7%; and arbidol, 43.9%). The complications included respiratory failure (26 [7.9%] patients), and acute respiratory distress syndrome (4 [1.2%] patients). 20 (6.1%) patients were admitted to the ICU. However, no patient died in our study (Table 3) [15].

**Table 3:** Treatment and outcomes of patients with coronavirus disease 2019

Variables (n [%])	All patients (n = 328)
Oxygen therapy	203 (61.9)
Mechanical ventilation	15 (4.6)
*Drug treatment*	
Atomized inhalation of interferon α-2b	181 (55.2)
Lopinavir/ritonavir	245 (74.7)
Arbidol	144 (43.9)
Antibiotic	256 (78.0)
Glucocorticoid	84 (25.6)
Gamma globulin	44 (13.4)
*Complications*	
Respiratory failure	26 (7.9)
ARDS	4 (1.2)
*Outcome*	
Severe illness	35 (10.7)
Admission to ICU	20 (6.1)
Death	0

*PCR* polymerase chain reaction, *ARDS* acute respiratory distress syndrome, *ICU* intensive care unit

### Associated factors of SARS-CoV-2 clearance

The associated factors of SARS-CoV-2 clearance in COVID-19 patients were analyzed by cox regression analysis (Table 4). The univariate analysis showed that the factors for patients with delayed clearance of SARS-CoV-2 were age > 60 years (hazard ratio [HR], 0.610, 95% confidence interval [CI] (0.450, 0.826), *P*  = 0.001), while the atomized inhalation of interferon α-2b (HR, 1.355, 95% CI 1.088, 1.688, *P* = 0.007) could improve the clearance of SARS-CoV-2. On the multivariate analysis, atomized inhalation of interferon α-2b (HR, 1.357, 95% CI 1.050–1.755, *P* = 0.020) was associated with an increased improvement of SARS-CoV-2 clearance compared with patients not receiving atomized inhalation of interferon α-2b, while age > 60 years (HR, 0.643, 95% CI 0.454–0.911, *P* = 0.013) was associated with a prolonged time to viral clearance compared with patients ≤ 60 years by adjusting sex, BMI, LDH, hypertension, and diabetes.

**Table 4:** Cox regression analysis of factors for the clearance of SARS-CoV-2 using date of initial symptoms as the start of follow-up

Variables	Univariate		Multivariate	
	HR (95% CI)	*P* value	HR (95% CI)	*P* value
Age (year)				
≤ 60	Reference			
> 60	0.610 (0.450, 0.826)	0.001	0.643 (0.454, 0.911)	0.013
Sex				
Female	Reference			
Male	0.992 (0.798, 1.233)	0.942	0.916 (0.721, 1.165)	0.475
BMI (kg/m^2^)				
< 28	Reference			
≥ 28	1.049 (0.751, 1.466)	0.778	1.155 (0.817, 1.631)	0.415
Hypertension				
No	Reference			
Yes	0.743 (0.549, 1.006)	0.055	0.905 (0.646, 1.269)	0.564
Diabetes				
No	Reference			
Yes	0.764 (0.515, 1.134)	0.181	0.869 (0.561, 1.346)	0.528
Lymphocytes				
No decreased	Reference			
Decreased	0.885 (0.698, 1.123)	0.316		
ALT (U/L)				
≤ 40	Reference			
> 40	1.057 (0.801, 1.396)	0.696		
LDH (U/L)				
≤ 250	Reference			
> 250	0.870 (0.697, 1.086)	0.217	0.935 (0.722, 1.210)	0.607
ALB (g/L)				
> 35	Reference			
≤ 35	0.774 (0.557, 1.075)	0.126		
D-dimer (mg/L)				
≤ 0.5	Reference			
> 0.5	0.857 (0.625, 1.176)	0.340		
Atomized inhalation of interferon α-2b				
No	Reference			
Yes	1.355 (1.088, 1.688)	0.007	1.357 (1.050, 1.755)	0.020
Lopinavir/ritonavir treatment				
No	Reference			
Yes	0.993 (0.771, 1.279)	0.957		

*PCR* polymerase chain reaction, *ALT* alanine transaminase, *LDH* lactate dehydrogenase, *ALB* albumin

**Fig. 3:**
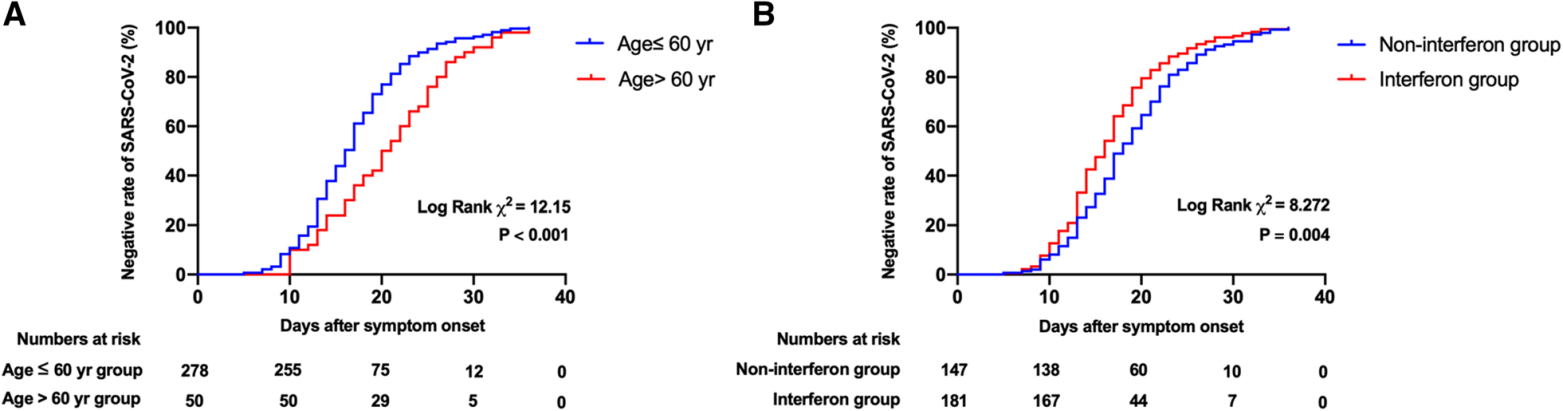
The cumulative clearance of SARS-CoV-2 in different age and treatment groups using date of initial symptoms as the start of follow-up

Accumulative clearance of SARS-CoV-2 was presented in the Fig. 3. The cumulative clearance of SARS-CoV-2 was significantly higher in age ≤ 60 years group than age > 60 years group (Log Rank χ^2^ = 12.15, *P* < 0.001) (Fig. 3a). The cumulative clearance of SARS-CoV-2 was significantly higher in interferon group compared to non-interferon group (Log Rank χ^2^ = 8.272, *P* = 0.004) (Fig. 3b).

As a sensitivity analysis, we further analyzed the associated factors of SARS-CoV-2 clearance using date of hospital admission as the start of follow-up. The results revealed that atomized inhalation of interferon α-2b (HR, 1.610, 95% CI 1.210–2.142, *P* = 0.001) could promote the clearance of SARS-CoV-2 compared with patients not treated with atomized inhalation of interferon α-2b, while age > 60 years (HR, 0.630, 95% CI 0.445–0.890, *P* = 0.009) was associated with a prolonged time to SARS-CoV-2 clearance compared with patients ≤ 60 years (Additional file 1: Table S1). Accumulative clearance of SARS-CoV-2 was presented in the Additional file 1: Fig. S1. The cumulative clearance of SARS-CoV-2 was significantly higher in age ≤ 60 years group than age > 60 years group (Log Rank χ^2^ = 18.89, *P* < 0.001) (Additional file 1: Fig. S1A). The cumulative clearance of SARS-CoV-2 was significantly higher in interferon group compared to non-interferon group (Log Rank χ^2^ = 12.23, *P* < 0.001) (Fig. S1B).

The comparisons of clinical characteristics between interferon group and non-interferon group were performed in the Additional file 1: Table S2. The results showed that more patients developed respiratory failure (12.9% vs. 3.9%, *P* = 0.003) and were admitted to ICU (10.2% vs. 2.8%, *P* = 0.005) in non-interferon group than interferon group. However, the demographic characteristics, laboratory and radiology findings were comparable between these two groups.

## Discussion

Our study described the clinical characteristics of 328 laboratory-confirmed cases of COVID-19 in 10 cities of Jiangsu province, China. Consistent with previous studies, fever and cough are the most common symptoms of COVID-19 in our study [4, 5]. Over 90% of the patients had abnormal chest CT results and ground-glass opacities are the most common findings in chest CT images. As of March 10, 2020, all patients were discharged, and no patient died in our study. The clinical outcomes of patients in our study are better than early cases reported in Wuhan with the fatality rate of 11.0–23.8% [7, 16]. In the summary of a report of 72, 314 Cases from the Chinese Center for Disease Control and Prevention, the overall case-fatality rate of COVID-19 was 2.3% [3]. Yang et al. reported 52 critically ill adult patients with SARS-CoV-2 pneumonia admitted to the ICU of Wuhan and the 28-day mortality rate is as high as 61.5% [17]. However, no deaths were reported among mild and severe cases [3]. In our study, only 13 (5.4%) patients were admitted to the ICU while all patients were recovered after treatment. The possible interpretation of the favorable outcomes in our study may be that the patients were younger and had less comorbidities than previous reports. Previous studies have demonstrated that elder and with more comorbidities were risk factors of admission to ICU and deceased [6, 18]. These results suggested that age and comorbidity may be risk factors for poor outcome. Another potential explanation of this different outcomes is the lack of medical resources in Wuhan during the outbreak of diseases due to the larger number of patients. However, in Jiangsu, there are enough medical facilities and less patients. Thus, readily available medical care could be easily accessed by and provided to SARS-CoV-2 infected patients which may have attributed to the reduced mortality of COVID-19 patients in Jiangsu.

The assessment of virus replication duration is important for evaluating the risk of transmission and guiding the isolation of patients. However, the duration of SARS-CoV-2 infection has not been well characterized. Zhou et al. reported that the detectable SARS-CoV-2 RNA persisted for a median of 20 days in survived COVID-19 patients and it was sustained in non-survivors of COVID-19 until death in Wuhan [7]. Our study found that the detectable SARS-CoV-2 RNA persisted for a median of 17 days which is shorter than patients reported in Wuhan (median 20.0 days).

The factors of viral clearance were analyzed in our study. We found that older age was associated with delayed clearance of SARS-CoV-2. Previous studies also found that older age was associated with prolonged SARS-CoV-2 RNA shedding [19, 20]. This may be explained by alteration of immune system related to age and ageing that could manifest as reduced immunity and defense to infection [21]. Therefore, more attention should be paid to older patients. In addition, atomized inhalation of interferon α-2b could improve viral clearance. Currently, no specific treatment is available for COVID-19. Although most of the patients received antiviral therapy including atomized inhalation of interferon α-2b, lopinavir/ritonavir and arbidol. The efficacy of these antiviral agents for COVID-19 is not yet clear. In the previous study, lopinavir/ritonavir treatment was not observed to shorten the clearance of SARS-CoV-2 [7]. Our study also confirmed that lopinavir/ritonavir treatment was not associated with the clearance of SARS-CoV-2. A randomized, controlled, open-label trial enrolled 99 COVID-19 patients with lopinavir-ritonavir group, and 100 COVID-19 patients with standard-care group has also found that the proportion of patients with detectable SARS-CoV-2 RNA were comparable at various time points. In our study, the arbidol treatment was not found to be associated with the clearance of SARS-CoV-2. Another study also reported that arbidol could not accelerate the SARS-CoV-2 clearance [22]. However, the results should be confirmed in larger prospective randomized studies. Atomized inhalation of interferon α-2b is recommended for the treatment of COVID-19 in the guidelines for COVID-19 by the Chinese National Health Commission [23]. Previous study also reported the effectiveness of interferon α-2b in middle east respiratory syndrome coronavirus infection [24, 25]. In COVID-19 patients, previous study also reported that inhaled interferon accelerated the clearance of SARS-CoV-2 [26, 27]. A retrospective study which enrolled 77 COVID-19 patients revealed that treatment with atomized inhalation of interferon α-2b significantly reduced the duration of detectable virus in COVID-19 patients [27]. However, the data in our study was from the beginning of the COVID-19 outbreak, and SARS-CoV-2 variant was rare at that time. Recently, numerous studies reported SARS-CoV-2 variant was common in new cases of COVID-19 [28–31]. These studies demonstrated that mutated SARS-CoV-2 spreads faster and may be more virulent [28, 29]. Therefore, the improved viral clearance by atomized inhalation of interferon α-2b as demonstrated in our study need to be confirmed in SARS-CoV-2 variant cohort.

This study has several limitations. First, only 328 patients with confirmed COVID-19 were included in the studies from 10 cities of Jiangsu province. We could not include all COVID-19 patients. However, the patients were included from 10 cities of Jiangsu province (54.2%). Thus, our study is representative of hospitalized cases in Jiangsu province. Secondly, there was potential ascertainment bias due to the fact that the interval between serial specimens for SARS-CoV-2 testing was not scheduled or standardized in our study. This is a retrospective study and no specific study protocol had been designed for scheduled specimen collection for SARS-CoV-2 testing, which were collected according to clinical condition as assessed by clinicians for clinical management during hospitalization. We cannot exclude the possibility that certain groups of patients would have had specimens collected at more frequent intervals that would have increased chance of having specimens with positive results. Moreover, data on clearance of SARS-CoV-2 might be influenced by the time point in the patients’ infection given the time of symptoms onset was reported by patients and recall bias might exist. Furthermore, the retrospective study design does not allow for causation to be determined with regards to the factors associated with the clearance of SARS-CoV-2 in COVID-19 patients. Finally, the association between clearance of SARS-CoV-2 and fatal outcome of patients could not be analyzed.

## Conclusions

The median duration of viral clearance after COVID-19 symptom onset was 17.0 days. Age > 60 years was associated with delayed clearance of while inhalation of atomized interferon alpha-2b hastened clearance of SARS-CoV-2. However, whether atomized inhalation of interferon α-2b may improve the clinical outcomes and reduce the death of COVID-19 deserve further investigation.

## Supplementary Information

**Additional file 1** **Fig. S1** The cumulative clearance of SARS-CoV-2 in different age and treatment groups using date of hospital admission as the start of follow up. **Table S1.** Cox regression analysis of factors for the clearance of SARS-CoV-2 using date of hospital admission as the start of follow-up. **Table S2.** Clinical characteristics of COVID-19 patients treated with interferon and without interferon.

## Data Availability

The data that support the findings of this study are available from the corresponding author upon reasonable request.

## Abbreviations

COVID-19: Coronavirus disease 2019
SARS-CoV-2: Severe acute respiratory syndrome coronavirus 2
RT-PCR: Real-time reverse transcription polymerase chain reaction
ARDS: Acute respiratory distress syndrome
IQR: Interquartile range
BMI: Body mass index
