# Supplementary material for: Clinical and virological course of patients with coronavirus disease 2019 in Jiangsu province, China: a retrospective, multi-center cohort study
Source: Virol J. 2021 Jul 14;18:147. doi: 10.1186/s12985-021-01615-y (PMC8278180; doi:10.1186/s12985-021-01615-y)
Supplement: Supplementary file 1 — Additional file 1 Fig. S1 The cumulative clearance of SARS-CoV-2 in different age and treatment groups using date of hospital admission as the start of follow up. Table S1. Cox regression analysis of factors for the clearance of SARS-CoV-2 using date of hospital admission as the start of follow-up. Table S2. Clinical characteristics of COVID-19 patients treated with interferon and without interferon. [file 12985_2021_1615_MOESM1_ESM.docx]

**Fig. S1 The cumulative clearance of SARS-CoV-2 in different age and treatment groups using date of hospital admission as the start of follow-up.**


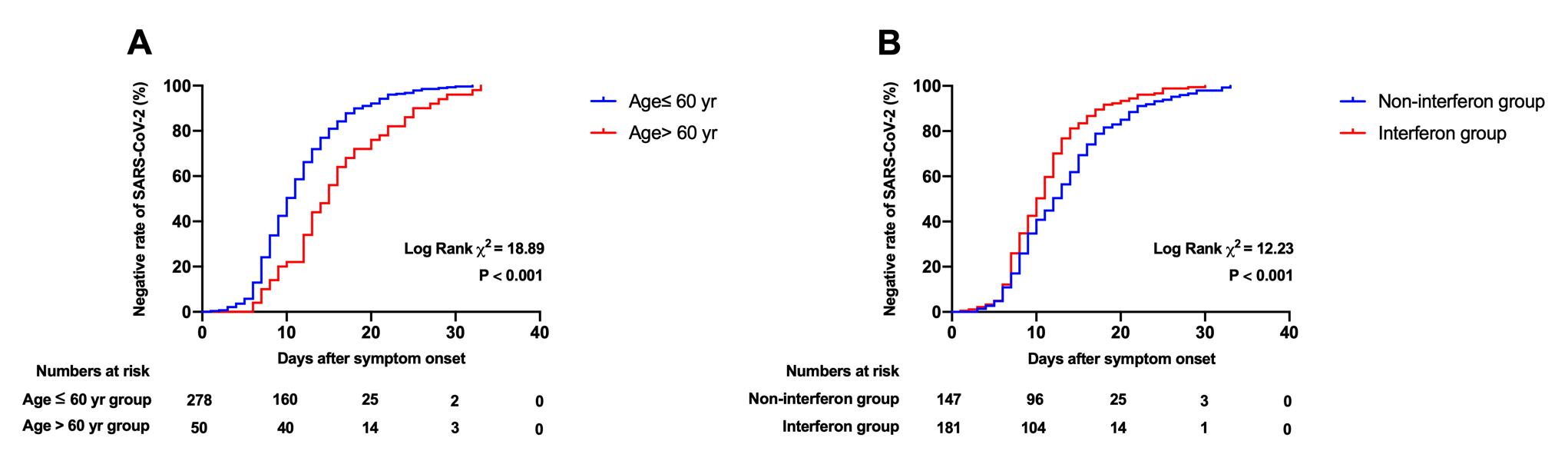


**Table S1. Cox regression analysis of factors for the clearance of SARS-CoV-2 using date of hospital admission as the start of follow-up.**

| Variables | Univariate | | | | Multivariate | |
| --- | --- | --- | --- | --- | --- | --- |
|  | HR (95% CI) | P value |  | HR (95% CI) | | P value |
| Age (yr) |  |  |  |  | |  |
| ≤60 | Reference |  |  |  | |  |
| >60 | 0.534 (0.392, 0.728) | <0.001 |  | 0.630 (0.445, 0.890) | | 0.009 |
| Sex |  |  |  |  | |  |
| Female | Reference |  |  |  | |  |
| Male | 1.112 (0.893, 1.385) | 0.342 |  | 1.036 (0.811, 1.323) | | 0.779 |
| BMI (kg/m^2^) |  |  |  |  | |  |
| <28 | Reference |  |  |  | |  |
| ≥28 | 0.872 (0.624, 1.218) | 0.423 |  | 0.914 (0.641, 1.303) | | 0.619 |
| Hypertension |  |  |  |  | |  |
| No | Reference |  |  |  | |  |
| Yes | 0.631 (0.464, 0.859) | 0.003 |  | 0.713 (0.507, 1.002) | | 0.051 |
| Diabetes |  |  |  |  | |  |
| No | Reference |  |  |  | |  |
| Yes | 0.636 (0.428, 0.944) | 0.025 |  | 0.805 (0.513, 1.263) | | 0.346 |
| Lymphocytes |  |  |  |  | |  |
| No decreased | Reference |  |  |  | |  |
| Decreased | 0.813 (0.642, 1.030) | 0.086 |  | 0.831 (0.621, 1.112) | | 0.213 |
| ALT (U/L) |  |  |  |  | |  |
| ≤40 | Reference |  |  |  | |  |
| >40 | 1.126 (0.852, 1.488) | 0.403 |  |  | |  |
| LDH (U/L) |  |  |  |  | |  |
| ≤250 | Reference |  |  |  | |  |
| >250 | 0.925 (0.742, 1.154) | 0.491 |  | 1.043 (0.793, 1.373) | | 0.762 |
| ALB (g/L) |  |  |  |  | |  |
| >35 | Reference |  |  |  | |  |
| ≤35 | 0.958 (0.690, 1.331) | 0.799 |  |  | |  |
| D-dimer (mg/L) |  |  |  |  | |  |
| ≤0.5 | Reference |  |  |  | |  |
| >0.5 | 0.912 (0.665, 1.251) | 0.567 |  |  | |  |
| Atomized inhalation of interferon α-2b | |  |  |  | |  |
| No | Reference |  |  |  | |  |
| Yes | 1.447 (1.159, 1.808) | 0.001 |  | 1.610 (1.210, 2.142) | | 0.001 |
| Lopinavir/ritonavir treatment |  |  |  |  | |  |
| No | Reference |  |  |  | |  |
| Yes | 0.767 (0.596, 0.986) | 0.038 |  | 0.768 (0.585, 1.007) | | 0.056 |

PCR, polymerase chain reaction; ALT, alanine transaminase; LDH, lactate dehydrogenase; ALB, albumin.

**Table S2**. **Clinical characteristics of COVID-19 patients treated with interferon and without interferon.**

| **Variables (n [%] or median [IQR])** | **Non-interferon group (n=147)** | **Interferon group (n=181)** | **P value** |
| --- | --- | --- | --- |
| Age (yr) | 44.0 (33.0, 55.0) | 47.0 (33.0, 55.5) | 0.714 |
| Male | 78 (53.1) | 98 (54.1) | 0.845 |
| BMI (kg/m^2^) | 24.2 (21.8, 26.1) | 24.2 (22.5, 26.3) | 0.596 |
| Hypertension | 16 (10.9) | 34 (18.8) | 0.048 |
| Diabetes | 9 (6.1) | 18 (9.9) | 0.210 |
| WBC (×10^9^/L) | 4.9 (3.9, 6.2) | 4.8 (3.8, 5.9) | 0.190 |
| Lymphocyte (×10^9^/L) | 1.2 (1.0, 1.7) | 1.2 (0.8, 1.6) | 0.070 |
| ALT (U/L) | 28.0 (21.8, 38.0) | 23.2 (16.9, 32.0) | 0.002 |
| Chest CT |  |  | 0.596 |
| No pneumonia | 12 (8.2) | 12 (6.6) |  |
| Pneumonia | 135 (91.8) | 169 (93.4) |  |
| Fever | 105 (71.4) | 132 (72.9) | 0.763 |
| Cough | 95 (64.6) | 105 (58.0) | 0.222 |
| Lopinavir/ritonavir | 96 (65.3) | 149 (82.3) | <0.001 |
| Arbidol | 60 (40.8) | 84 (46.4) | 0.310 |
| Respiratory failure | 19 (12.9) | 7 (3.9) | 0.003 |
| ARDS | 1 (0.7) | 3 (1.7) | 0.423 |
| Severe illness | 21 (14.3) | 14 (7.7) | 0.056 |
| Admission to ICU | 15 (10.2) | 5 (2.8) | 0.005 |

IQR, interquartile range; BMI, body mass index; WBC, white blood cells; Hb, hemoglobin; ALT, alanine transaminase; PCR, polymerase chain reaction; ARDS, acute respiratory distress syndrome; ICU, intensive care unit.
